# Trait Intolerance of Uncertainty Is Associated with Decreased Reappraisal Capacity and Increased Suppression Tendency

**DOI:** 10.1007/s42761-022-00115-8

**Published:** 2022-06-03

**Authors:** Jocelyn Shu, Kevin N. Ochsner, Elizabeth A. Phelps

**Affiliations:** 1 Department of Psychology, Harvard University, 52 Oxford St., Cambridge, MA 02138 USA; 2 Department of Psychology, Columbia University, New York, NY USA

**Keywords:** Intolerance of uncertainty, Emotion regulation, Reappraisal, Suppression, Worry

## Abstract

The COVID-19 pandemic has been a time of great uncertainty for the general population and highlights the need to understand how attitudes towards uncertainty may affect well-being. Intolerance of uncertainty is a trait associated with worry, anxiety, and mood disorders. As adaptive emotion regulation supports well-being and mental health, it is possible that intolerance of uncertainty is also associated with the ability and tendency to regulate emotions. However, the relationships between intolerance of uncertainty and widely studied cognitive emotion regulation strategies — such as reappraisal and suppression — have received little attention. In two studies that recruited participants online from the United States, we tested the hypotheses that higher trait intolerance of uncertainty would be associated with greater worry, decreased capacity and tendency to use reappraisal, and increased tendency to use suppression in daily life. Study 1 provided an initial test of our hypotheses. Study 2 was a confirmatory, preregistered study that replicated findings in a young adult sample, demonstrating that scores on the Intolerance of Uncertainty Scale (IUS) were associated with greater COVID-related worry, decreased capacity to regulate negative emotions on a task that manipulated the use of reappraisal, and greater self-reported use of suppression in daily life. Together, these results indicate that intolerance of uncertainty is associated with the capacity and tendency to use emotion regulation strategies important for well-being.

**Supplementary Information:**

The online version contains supplementary material available at 10.1007/s42761-022-00115-8.

For some individuals, the uncertainty inherent in not knowing how future events may unfold is experienced as aversive and can contribute to anxiety and mood disorders (Birrell, 2011; Tanovic et al., 2018). This is of particular importance during the COVID-19 pandemic — which has caused people to experience high levels of uncertainty. Critically, uncertain times are likely to be more difficult for individuals with high trait intolerance of uncertainty. Indeed, research conducted during the pandemic has demonstrated intolerance of uncertainty to be associated with increased distress (Bavolar et al., 2021; Rettie & Daniels, 2020; White, 2022). Given the relationship between distress and emotion regulation, individuals who are intolerant of uncertainty may be predisposed to not only experience greater anxiety but also to have greater difficulty regulating their emotions. However, little research has investigated the relationship between intolerance of uncertainty and emotion regulation.

Intolerance of uncertainty can be manipulated as a state (Ladouceur et al., 2000) or assessed as an enduring trait — the latter being commonly measured with the Intolerance of Uncertainty Scale (IUS; Birrell, 2011; Buhr & Dugas, 2002). The IUS measures self-reported attitudes towards uncertainty and the degree to which intolerance of uncertainty is believed to hinder behavior. Research has established intolerance of uncertainty to be associated with a host of affective disorders, including generalized anxiety disorder (GAD) and depression (Carleton et al., 2012; Gentes & Ruscio, 2011; Tanovic et al., 2018). IUS also has been demonstrated to be associated with self-perceived difficulties with emotion regulation (Ouellet, 2019). However, to our knowledge, prior research has not assessed the relationship between intolerance of uncertainty and the capacity and tendency to implement cognitive emotion regulation strategies.

Two types of findings may inform our thinking about the relationships between IUS and emotion regulation. First, research has found that intolerance of uncertainty impedes threat extinction learning (Dunsmoor, Campese, et al., 2015; Morriss et al., 2016, 2019). Threat extinction learning is the process by which one learns that a stimulus formerly associated with a threat is now safe (Dunsmoor, Niv, et al., 2015). It is a form of emotion regulation that can happen without deliberate effort and is impaired in individuals with anxiety disorders (Craske et al., 2018; Dunsmoor, Niv, et al., 2015). IUS scores have been associated with slowed extinction to threat cues (Morriss et al., 2016, 2019) and with more spontaneous recovery of learned threat responses (Dunsmoor, Campese, et al., 2015).

Second, research indicates that intolerance of uncertainty may increase behaviors considered to be maladaptive forms of emotion regulation, such as worry — a defining feature of GAD (Buhr & Dugas, 2002; Ladouceur et al., 2000). Indeed, intolerance of uncertainty has been demonstrated to be a causal driver of worry — a cognitive process by which thoughts about potential threats are rehearsed uncontrollably (Ladouceur et al., 2000). Worry may represent attempts to reduce uncertainty and suppress anxiety-related somatic responses (Borkovec et al., 2004). However, it can lead instead to catastrophizing potential outcomes (Freeston et al., 1994; Tanovic et al., 2018). IUS has been associated with more negative appraisals, and worry has been found to partially mediate this relationship (Koerner & Dugas, 2008).

Here, we sought to investigate how intolerance of uncertainty may be associated with two widely studied cognitive emotion regulation strategies — reappraisal and suppression. Reappraisal involves changing one’s interpretation of a stimulus to alter one’s emotional response to it. By contrast, suppression limits the behavioral expression of emotion (Gross & John, 2003; Gross & Levenson, 1993). Reappraisal is generally thought to have longer-lasting effects as it modifies the representation of the emotion eliciting stimulus. Suppression, however, does not decrease the experience of negative emotions as much as reappraisal as it aims to mitigate the expression, as opposed to the experience, of the emotion (Gross, 1998, 2002). That said, context determines whether reappraisal and suppression are adaptive or maladaptive (Butler et al., 2007; Doré et al., 2016; Ford & Troy, 2019; Troy et al., 2013). Although generally thought to be beneficial, reappraisal can have detrimental effects when implemented unsuccessfully (Ford & Troy, 2019) and is differentially preferred depending on factors such as the intensity and type of emotion experienced, as well as the controllability of one’s situation (Sheppes et al., 2011; Shu et al., 2021; Troy et al., 2013). On the other hand, the ability to use suppression flexibly is associated with better psychological outcomes (Bonanno et al., 2004), and the negative effects of suppression can be moderated by cultural values (Butler et al., 2007).

Reappraisal and suppression are typically studied in two ways: the capacity (i.e., ability) to implement the strategy, and the tendency to use it (Silvers & Guassi Moreira, 2019). In the lab, reappraisal capacity is often studied using tasks that present participants with aversive stimuli (i.e., photographic images) and instruct them to either respond naturally (baseline condition) or reappraise the stimuli (Denny & Ochsner, 2014; McRae, Gross, et al., 2012; Silvers et al., 2016). Critically, this task structure allows for separate assessments of (1) bottom-up affective responding in the baseline condition, and (2) top-down regulatory control in the reappraisal condition. This is important as emotional experiences can result from either process, each of which recruits distinct neural regions with differing implications for theoretical and clinical understanding (Buhle et al., 2014; Ochsner et al., 2002).

Reappraisal capacity and tendency are distinct constructs that can be weakly related to each other (McRae, Jacobs, et al., 2012; Silvers & Guassi Moreira, 2019; Troy et al., 2010). The trait tendencies to use reappraisal and suppression in daily life are often assessed using the Emotion Regulation Questionnaire (ERQ), which measures self-reported use of these strategies (Gross & John, 2003). As such, a fuller understanding of how intolerance of uncertainty relates to emotion regulation would involve assessing how intolerance of uncertainty is associated with both the capacity and tendency to employ emotion regulation strategies.

As stress impacts the prefrontal cortex, which is crucial for exerting cognitive control (Arnsten, 2009), it can also impair reappraisal (Raio et al., 2013). Thus, we might expect intolerance of uncertainty, and the consequent worry and stress it generates, to also impair reappraisal. It may be that as individuals who are more intolerant of uncertainty experience increased stress and worry, thus becoming less capable of using reappraisal, they resort to suppressing their negative emotions. Recruiting participants online during COVID-19 lockdowns, we hypothesized that trait intolerance of uncertainty would be associated with (1) greater worry related to the pandemic, (2) reduced capacity and tendency to use reappraisal, and (3) increased tendency to use suppression. Given prior literature on the causal role of intolerance of uncertainty in worry (Ladouceur et al., 2000), we also hypothesized that COVID-related worry would mediate the relationship between intolerance of uncertainty and emotion regulation. As this research aims to investigate how individual differences in intolerance of uncertainty may be associated with important affective outcomes during the pandemic, we employed correlational methods to test the relationships between IUS and emotion regulation. To assess reappraisal capacity, we adapted a commonly used reappraisal task (e.g., Denny & Ochsner, 2014; McRae, Jacobs, et al., 2012; Ochsner et al., 2002; Silvers et al., 2016) so that it could be administered online. If intolerance of uncertainty is associated specifically with reduced capacity for reappraisal, IUS should be associated with greater negative affect when using reappraisal, but not when responding naturally to negative stimuli. We did not assess the capacity to implement suppression as we sought to study associations with the capacity for emotion regulation generally thought to be beneficial. To assess emotion regulation tendencies, we modified the ERQ to assess *recent,* instead of trait, use of reappraisal and suppression in daily life. Study 1 was an initial test of our hypotheses. Study 2 was a preregistered, confirmatory study in which we addressed potential design issues from Study 1 and replicated certain findings.

## Study 1

### Participants

Two hundred participants from the United States were recruited on Prolific from May 18–22, 2020. This occurred during the initial COVID-19 lockdowns implemented throughout the country. Participant recruitment was restricted to those residing in the United States with an approval rating of at least 95%, who had completed at least 10 other studies on Prolific, and who were working from a desktop computer. Participants were excluded from analyses if they made more than four attempts to answer three comprehension questions prior to starting the emotion regulation task, or if they did not pass an attention check (Oppenheimer et al., 2009) at the end of the study. The recruitment number was determined *a priori* as being likely to detect an existing effect for small to medium effect sizes after excluding participants who do not fulfill the criteria for inclusion in analyses. A power analysis was not conducted due to the initial, exploratory nature of this study and uncertainty in determining an effect size. The final sample consisted of 163 participants (*M*_Age_ = 31.7, *SD* = 11.33, range = 18–74; gender: 68 female, 93 male, 2 other). A sensitivity analysis using G*Power v. 3.1.9.5, for our primary analysis assessing the relationship between IUS and reappraisal capacity using multiple linear regression, indicated this final sample size as being able to reliably detect a small effect size of *f*^2^ = .049 (*α* = .05,power = .8, number of tested predictors = 1, number of predictors = 4).

### Method

#### Reappraisal Task Training

All procedures were approved by the Harvard University Institutional Review Board. Participants received a link to the experiment created and hosted on Gorilla (www.gorilla.sc; Anwyl-Irvine et al., 2020). They first completed a bot check and consent form, followed by a training session in which they read through instructions for the emotion regulation task. There were three conditions in this task: Reappraise, Look Negative, and Look Neutral. While the Reappraise Condition was of primary interest, the Look Negative condition served as a control condition to assess emotional reactivity. Studies employing a similar version of this reappraisal task typically assess reappraisal capacity by accounting for emotional reactivity as measured by the Look Negative Condition or other similar conditions (Buhle et al., 2014). The Look Neutral condition is another baseline condition but primarily serves to provide filler trials to mitigate habituation to negative stimuli.

The training session consisted of a series of instructions that the participant read through. These instructions first walked the participant through what to do when presented with instruction cues for the *Look* and *Reappraise* conditions. The Look conditions consisted of the Look Negative and Look Neutral conditions. For the Look conditions, participants were told that one instruction cue would tell them to “Look at the image,” and that they should respond naturally to the upcoming image (either negative or neutral) when this cue was presented. For the Reappraise Condition, participants were told that the other instruction cue would tell them to “Reframe the image” and that when they saw this cue, they should rethink the meaning of the upcoming image in a way that would decrease their negative emotions. The instructions suggested that in order to do this, they could try to look on the bright side by finding positive aspects of the depicted situation, or by thinking about how some aspects of the situation may not be as bad as they may seem to be. Reappraise trials always consisted of a negative image.

**Fig. 1:**
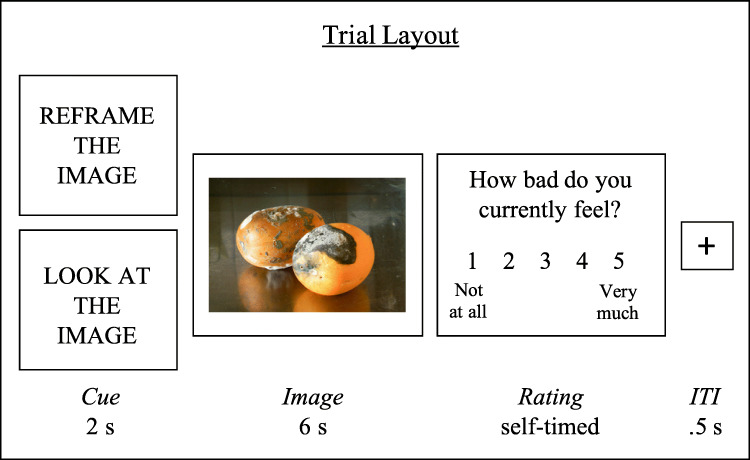
Trial layout

After reading through these instructions, participants were shown example images and told that they would rate their emotions after viewing the picture. Participants were told that when rating their emotions, they should rate how they were feeling at the moment, regardless of how effectively they had implemented the instruction from the cue. Participants were then asked three questions with multiple-choice answers that quizzed them on their comprehension of these instructions. They received automated feedback on the accuracy of their responses and were required to answer correctly before proceeding. The training session then presented examples to participants of each stage within a trial in the emotion regulation task (see Fig. 1 for trial layout). Participants completed six practice trials (three for the Reappraise and three for the Look conditions), and after each practice trial, they were asked to write out what they had been thinking when viewing the image for that trial. These responses were checked by the experimenter after the participant completed the study to ensure the participant was engaging in the training session and understood the instructions. After completing the practice trials, participants started the reappraisal task.

#### Reappraisal Task

The task consisted of two runs with 30 trials in each run (20 trials/condition across both runs). Trials were grouped into blocks of three, with each block consisting of one trial from each of the three conditions (Reappraise, Look Negative, Look Neutral). The order of trials was randomized within each block, and blocks were randomly ordered within each run.

Stimuli were obtained from the Disgust-Related-Images (DIRTI) database. Negative images depicted scenes of injuries, dead animals, bodily waste, and spoiled food, whereas neutral images included scenes of fresh food, nature, household appliances, and peaceful scenes of humans and animals (Haberkamp et al., 2017). We used this stimulus set because it is allowed for use in online studies administered over platforms such as Prolific and contains similar content to stimulus sets commonly used in lab-based studies on emotion and emotion regulation (e.g., Lang et al., 1997). We constructed three sets of images (40 negative and 20 neutral images in each set) with similar mean normed affect ratings across sets (Negative: *M* = 3.14, *SD* = .34; Neutral: *M* = 7.23, *SD* = .74; 1 = very negative, 5 = neutral, 9 = very positive). Out of these sets, we created further subsets so that negative images used for the Reappraise and Look Negative conditions were exchanged across subsets. Participants were presented with one image set during the task, with image sets randomly assigned to participants. Each set was split in half for the two runs and the run order was randomized for each participant.

#### Questionnaires

After completing the emotion regulation task, participants completed questionnaire measures administered with Qualtrics. We administered the following measures: Intolerance of Uncertainty Scale (IUS), a modified version of the Emotion Regulation Questionnaire (ERQ), Marlowe-Crowne Social Desirability Scale short form (MCSD), Positive and Negative Affect Schedule (PANAS), Perceived Stress Scale (PSS), and the Depression, Anxiety, & Stress Scale (DASS-21). These questionnaires were administered in random order. Following the questionnaires, participants completed a lab-constructed survey designed to assess various aspects of their thoughts, emotions, and experiences with the COVID-19 pandemic. A measure of COVID-related worry was constructed with items from this survey. After the survey, participants provided demographics information, completed an attention check (Oppenheimer et al., 2009), and were provided a list of mental health resources they could consult in case they were experiencing distress. Participants took about an hour to complete the study and were compensated $15.

The current analyses focused on the IUS, modified ERQ, and COVID-related worry as primary measures of interest. The MCSD was administered to adjust for social desirability effects. All measures were aggregated by calculating the mean score of items unless otherwise noted. The IUS is a 27-item measure that assesses how people respond to uncertainty with statements such as “Uncertainty makes me uneasy, anxious, or stressed” and “My mind can’t be relaxed if I don’t know what will happen tomorrow.” The IUS is rated on a 5-point Likert scale (1 = Not at all characteristic of me, 5 = Entirely characteristic of me, and has demonstrated good reliability; Buhr & Dugas, 2002).

The ERQ is a 10-item measure that assesses trait individual differences in the use of reappraisal (6 items; e.g., “When I want to feel less negative emotion, I change the way I’m thinking about the situation”) and suppression (4 items; e.g., “When I am feeling negative emotions, I make sure not to express them”) as emotion regulation strategies. The ERQ is rated on a 7-point Likert scale (1 = Strongly disagree, 7 = Strongly agree) and has demonstrated good reliability (Gross & John, 2003). Although the ERQ assesses trait use of reappraisal and suppression, we were concerned that during study recruitment — which occurred during major waves of lockdowns in the United States during the COVID-19 pandemic — the ERQ would instead reflect state-like tendencies due to stressors caused by the pandemic and its accompanying health and economic challenges. To explicitly assess current tendencies for using reappraisal and suppression in daily life, we modified the ERQ to assess state tendencies by asking participants to rate their responses according to how they have *recently* been managing their emotions.

To assess the current level of worry about the COVID-19 pandemic, we aggregated 24 items (see Table S1 for all items) that specifically assessed how worried participants were about issues related to the pandemic (e.g., the possibility of becoming infected with COVID, loss of employment) from the lab-constructed survey. These items were rated on a 5-point Likert scale (1 = Not at all worried, 3 = Somewhat worried, 5 = Extremely worried). Twenty-two items had an option to indicate N/A if the item was not relevant to the participant. Items answered with N/A were dropped and mean scores were calculated from the remaining items for each participant.

The MCSD short form is a 13-item measure that assesses how often participants endorse socially desirable descriptions of themselves (e.g., “I have never been irked when people expressed ideas very different from my own”). The items are rated on a true/false binary scale according to whether participants perceive these statements to be true of themselves, and the scores are summed according to whether the response reflects the motivation to provide a socially desirable response. This scale has demonstrated good reliability (Reynolds, 1982).

### Results

Data and analysis scripts can be accessed at https://osf.io/kpf3d. Analyses were conducted with R version 4.0.3 (R Core Team, 2020) and RStudio version 1.3.1093 (RStudio Team, 2020). Inferential statistics were performed with two-sided tests.

The following analyses test our key hypotheses concerning whether IUS is associated with COVID-related worry, impaired reappraisal, and increased suppression. Exploratory analyses were conducted to assess relationships between COVID-related worry and emotion regulation, and a mediating role for COVID-related worry in the relationships between IUS and emotion regulation. Analyses were adjusted for social desirability (MCSD) to account for potential biases from the use of desirable responding in self-report measures. Analyses were also adjusted for age because age-related differences in affect and well-being are often observed, including during the COVID-19 pandemic (Carstensen et al., 2011, 2020; Mather, 2012).

For correlation analyses throughout the manuscript, we used partial correlations to adjust for covariates. Pearson’s *r* correlations were performed between variables that were normally distributed and Spearman’s rho correlations were performed if a variable was not normally distributed (*p* < .05 on the Shapiro-Wilk normality test). Confidence intervals for partial correlations were calculated with bootstrapping using 10,000 samples. Exploratory analyses with other measures are reported in the SOM.

Given the exploratory nature of this study, we report uncorrected *p*-values along with Bonferroni corrected *p*-values for analyses assessing the relationships between IUS and emotion regulation, as well as between COVID-related worry and emotion regulation. For these analyses, we correct for three tests (*p* = .05/3) to account for the three kinds of emotion regulation strategies assessed (reappraisal capacity on the task, reappraisal tendency on ERQ, and suppression tendency on ERQ). Bonferroni corrected *p*-values are reported in parentheses next to uncorrected *p*-values.

#### Manipulation Check

A paired *t*-test indicated that as expected, mean negative affect in the Reappraise Condition (*M* = 2.64, *SD* = .70) was lower than in the Look Negative Condition, *M* = 3.17, *SD* = .81; *M*_Diff_ = −.53, 95% CI [−.62, −.45], *t*(162) = −12.57, *p* < .001, *d* = −.70. Mean negative affect was greater in the Reappraise Condition than in the Look Neutral Condition, *M* = 1.25, *SD* = .38; *M*_Diff_ = 1.39, 95% CI [1.29, 1.49], *t*(162) = 27.65, *p* < .001, *d* = 2.34, which is also expected as the two conditions consist of differently valenced stimuli.

#### IUS and COVID-Related Worry

As expected, IUS (*M* = 2.64, *SD* = .78, *α* = .95) was associated with greater COVID-related worry, *M* = 2.72, *SD* = .88, *α* = .95; *r*_s_(159) = .35, 95% CI [.20, .50], *p* < .001, when adjusting for MCSD (*M*_Sum_ = 5.43, *SD* = 2.96, *α* = .74) and age.

#### IUS and Reappraisal Capacity

To assess how IUS was associated with reappraisal capacity, we first assessed the relationship between IUS and mean negative affect in the Reappraise Condition of the task. As mean affect scores in the Reappraise and Look Negative conditions were correlated, *r*_*s*_(161) = .69, 95% CI [.60, .76], *p* < .001, and also because emotional reactivity is typically treated as a control condition in reappraisal studies, we adjusted for emotional reactivity (i.e., mean affect in Look Negative Condition), along with age and MCSD, to ensure that effects were specific to reappraisal. A multiple linear regression was performed with IUS as a predictor for mean affect ratings in the Reappraise Condition, with mean affect in the Look Negative Condition, MCSD scores, and age as covariates. IUS was a statistically significant predictor of increased negative affect in the Reappraise Condition at an uncorrected level, but this relationship was at trend level with Bonferroni correction (see Table 1).

**Table 1:** Study 1: Effect of predictors on mean negative affect in Reappraise Condition (*n* = 163)

Measure	*b* [95% CI]	*SE*	*t*	*p*	*f*^2^
IUS	.11 [.012, .22]	.051	2.22	.028 (.084)	.031
Look Neg Condition	.60 [.51, .70]	.046	13.08	<.001	1.08
MCSD	−.001 [−.027, .024]	.013	−.11	.91	.000
Age	−.006 [−.012, .0007]	.003	−1.77	.079	.020

Notes. Multiple linear regression results. *F*(4, 158) = 56.43, *p* < .001, adjusted *R*^*2*^ *=* .58. Bonferroni corrected *p*-value is reported for IUS in parentheses. *IUS* Intolerance of Uncertainty Scale, *Look Neg* mean negative affect in Look Negative Condition, *MCSD* Marlowe Crowne Social Desirability Scale

If intolerance of uncertainty is specific in its association with reappraisal, as opposed to emotional reactivity in general, then IUS scores should be associated with greater negative affect during the Reappraise Condition, but not during the Look Negative Condition. Thus, to further determine the specificity of the relationship between IUS and reappraisal capacity, we assessed the relationship between IUS and emotional reactivity on the reappraisal task, while adjusting for reappraisal capacity, MCSD scores, and age. A multiple linear regression was performed to assess whether IUS was associated with mean affect ratings in the Look Negative Condition when adjusting for affect in the Reappraise Condition, age, and MCSD scores. IUS scores were not significantly associated with negative affect in the Look Negative Condition when adjusting for these covariates (see Table 2; zero-order correlations between all measures are reported in Table S2).

**Table 2:** Study 1: Effect of predictors on mean negative affect in Look Negative Condition (*n* = 163)

Measure	*b* [95% CI]	*SE*	*t*	*p*	*f*^2^
IUS	.040 [−.083, .16]	.062	.65	.52	.003
Reappraise Condition	.86 [.73, .99]	.066	13.08	<.001	1.08
MCSD	.003 [−.028, .034]	.016	.20	.84	.000
Age	.002 [−.005, .010]	.004	.62	.54	.002

Notes. Multiple linear regression results. *F*(4, 158) = 50.68, *p* < .001, adjusted *R*^*2*^ *=* .55. *IUS* Intolerance of Uncertainty Scale, *Reappraise Condition* mean negative affect in Reappraise Condition, *MCSD* Marlowe Crowne Social Desirability Scale

#### IUS and Reappraisal Tendency

Then, to assess the relationship between IUS scores and reappraisal tendency, we performed a correlation analysis between IUS scores and the reappraisal subscale of the modified ERQ (*M* = 4.84, *SD* = 1.24, *α* = .90). Contrary to our predictions, after adjusting for MCSD and age, IUS scores were not significantly correlated with the recent use of reappraisal, as reported on the reappraisal subscale of the modified ERQ, *r*_s_(159)= −.11, 95% CI [−.27, .047], *p* = .17 (.51).

#### IUS and Suppression Tendency

IUS scores were significantly correlated with scores on the suppression subscale of the modified ERQ (*M* = 3.76, *SD* = 1.37, *α* = .81) when adjusting for MCSD and age, *r*_s_(159)= .32, 95% CI [.19, .47], *p* < .001 (.001).

#### Relationships between COVID-Related Worry and Emotion Regulation

We conducted exploratory analyses to assess whether COVID-related worry was associated with reappraisal capacity, reappraisal tendency, and suppression tendency. After adjusting for emotional reactivity on the task, MCSD scores, and age, COVID-related worry was not associated with reappraisal capacity on the task, *r*_*s*_(158) = .11, 95% CI [−.052, .29], *p* = .15 (.46), nor was COVID-related worry associated with reappraisal tendency on the modified ERQ, *r*_*s*_(159)= .037, 95% CI [−.13, .20], *p* = .64 (1). However, COVID-related worry was significantly associated with greater use of suppression on the modified ERQ when adjusting for MCSD scores and age, *r*_s_(159) = .25, 95% CI [.11, .40], *p* = .001 (.004).

#### Mediation Analysis

As IUS, COVID-related worry, and suppression tendency were correlated, we then assessed whether COVID-related worry mediated the relationship between IUS and suppression tendency. We performed a mediation analysis with bootstrapping using 10,000 samples adjusting for MCSD scores and age. This analysis indicated the presence of a mediating effect (*ab* = .12, 95% CI [.011, .24], *p* = .027, proportion mediated = .18, *p* = .027).

## Study 2

The aim of Study 2 was to replicate findings from Study 1. Preregistration for our hypotheses, target recruitment, and certain analyses (analyses are preregistered unless otherwise noted) can be accessed at https://aspredicted.org/zy7it.pdf. As age was associated with IUS, worry, and emotion regulation outcomes in Study 1, we restricted the age range of participants in this study to limit the potential confounding effect of age. Furthermore, as participants completed questionnaires after the reappraisal task in Study 1, responses on the questionnaires could have been affected by their performance on the reappraisal task. To account for potential order effects in this study, we counterbalanced the order that the questionnaires and reappraisal task were administered across participants (as in Study 1, the measure of COVID-related worry was administered following the other questionnaires). In addition to the questionnaires administered in Study 1, we also administered the Affective Lability Scale short form (ALS) and Penn State Worry Questionnaire (PSWQ) in this study (see SOM for exploratory analyses).

### Participants

Four-hundred and seventy-seven participants were recruited from Prolific from December 11 to 16, 2020, during the second wave of COVID-19 lockdowns implemented throughout the United States. The same recruitment restrictions in Prolific as in Study 1 were applied; except in this study, we restricted recruitment of participants to those between the ages of 18–35 in order to limit the effects of age. As in Study 1, participants were excluded from analyses if they made more than four attempts to answer the three comprehension questions prior to starting the reappraisal task, if they did not pass the attention check at the end of the study, if they did not provide a written narrative response of their experience during the pandemic as requested in the COVID survey, or if there was no variability in their responses on the emotion regulation task.

The final sample consisted of the target recruitment number of 400 participants (*M*_Age_ = 27.4, *SD* = 4.38, range = 18–35; Gender: 169 female, 226 male, 5 other). This recruitment number was based on a power analysis conducted in G*Power v. 3.1.9.5 for linear multiple regression. We based the power analysis on the effect from Study 1 indicating that IUS predicted reappraisal capacity on the emotion regulation task when adjusting for reactivity (Look Negative scores), MCSD scores, and age (*f*^2^ = .031, *α* = .05, power = .8, number of tested predictors = 1, number of predictors = 4). This analysis indicated a sample size of 256 participants to be necessary for detecting an existing effect. We used this analysis as a basis to decide *a priori* to recruit 400 participants who meet inclusion criteria to account for an actual effect size that may be smaller, or additional analyses that may require greater power.

### Method

Methods in this study were largely identical to those in Study 1. Participants were randomly assigned to different orders of the task and questionnaires, such that half the participants completed the reappraisal task first and half completed the questionnaires first. An attention check was administered after completing the questionnaires and demographics information was assessed at the end of the study. Participants took about an hour to complete the study and were compensated $15.

### Results

#### Manipulation Check

**Fig. 2:**
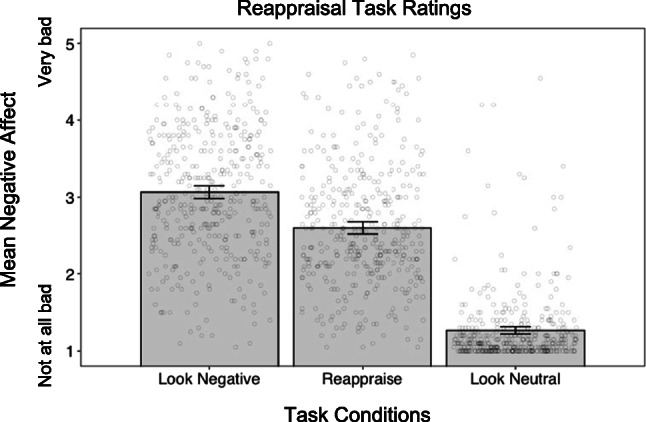
Reappraisal task ratings. Notes. Mean affect ratings from reappraisal task in Study 2. Error bars ± 95% CI

As expected, a paired *t*-test indicated that mean negative affect in the Reappraise Condition of the reappraisal task (*M* = 2.60, *SD* = .79) was lower than in the Look Negative Condition, *M* = 3.07, *SD* = .84; *M*_Diff_ = −.46, 95% CI [−.52, −.41], *t*(399) = −15.86, *p* < .001, *d* = −.56. Mean negative affect was greater in the Reappraise Condition than in the Look Neutral Condition, *M* = 1.27, *SD* = .47; *M*_Diff_ = 1.34, 95% CI [1.26, 1.41], *t*(399) = 33.69, *p* < .001, *d* = 1.99; see Fig. 2. The following analyses tested our key hypotheses and were identical to those in Study 1.

#### IUS and COVID-Related Worry

As predicted, IUS scores (*M* = 2.65, *SD* = .86, *α* = .96) were associated with greater degree of COVID-related worry, *M* = 2.82, *SD* = .81, *α* = .93; *r*_s_(396) = .37, 95% CI [.28, .47], *p* < .001, when adjusting for MCSD scores (*M* = 5.54, *SD* = 2.99, *α* = .75) and age.

#### IUS and Reappraisal Capacity

A multiple linear regression was performed to assess whether IUS scores predict mean affect ratings in the Reappraise Condition while adjusting for mean ratings in the Look Negative Condition, MCSD, and age. As expected, IUS was a statistically significant predictor of increased negative affect when using reappraisal (see Table 3). Also, as expected, multiple linear regression indicated that when adjusting for mean ratings in the Reappraise Condition, MCSD scores, and age, IUS scores were not associated with negative affect in the Look Negative Condition (see Table 4; zero-order correlations between all measures are reported in Table S3).

**Table 3:** Study 2: Effect of predictors on mean negative affect in Reappraise Condition (*n* = 400)

Measure	*b* [95% CI]	*SE*	*t*	*p*	*f*^2^
IUS	.077 [.013, .14]	.032	2.37	.019	.014
Look Neg Condition	.70 [.63, .76]	.031	22.14	<.001	1.24
MCSD	.002 [−.016, .02]	.009	.23	.82	.000
Age	−.008 [−.02, .004]	.006	−1.28	.20	.004

Notes. Multiple linear regression results. *F*(4, 395) = 129.3, *p* < .001, adjusted *R*^*2*^ *=* .56. *IUS* Intolerance of Uncertainty Scale, *Look Neg* mean negative affect in Look Negative Condition, *MCSD* Marlowe Crowne Social Desirability Scale

**Table 4:** Study 2: Effect of predictors on mean negative affect in Look Negative Condition (*n* = 400)

Measure	*b* [95% CI]	*SE*	*t*	*p*	*f*^2^
IUS	−.008 [−.076, .061]	.035	−.22	.83	.000
Reappraise Condition	.80 [.72, .87]	.036	22.14	<.001	1.24
MCSD	.006 [−.014, .025]	.010	.59	.56	.001
Age	.007 [−.006, .019]	.006	1.01	.31	.003

Notes. Multiple linear regression results. *F*(4, 395) = 125.6, *p* < .001, adjusted *R*^*2*^ *=* .56. *IUS* Intolerance of Uncertainty Scale, *Reappraise Condition* mean negative affect in Reappraise Condition, *MCSD* Marlowe Crowne Social Desirability Scale

#### IUS and Reappraisal Tendency

We assessed the relationship between IUS scores and reappraisal tendency. However, we did not preregister this analysis due to insignificant results when adjusting for covariates in Study 1. Unlike Study 1, there was a significant relationship between IUS scores and the reappraisal subscale of the modified ERQ (*M* = 4.95, *SD* = 1.13, *α* = .87) when adjusting for MCSD scores and age, *r*_s_(396) = −.16, 95% CI [−.27, −.064], *p* = .001.

#### IUS and Suppression Tendency

As predicted, IUS scores were significantly correlated with greater use of suppression as reported on the modified ERQ (*M* = 4.06, *SD* = 1.30, *α* = .75) when adjusting for MCSD scores and age, *r*_s_(396) = .14, 95% CI [.042, .24], *p* = .005.

#### Relationships Between COVID-Related Worry and Emotion Regulation

We conducted exploratory analyses that were not preregistered, examining whether COVID-related worry was associated with reappraisal capacity and tendency in this study. Unlike Study 1, COVID-related worry was associated with decreased reappraisal capacity on the task, when adjusting for Look Negative scores, MCSD scores, and age, *r*_*s*_(395) = .11, 95% CI [.009, .21], *p* = .030. As in Study 1, COVID-related worry was not associated with reappraisal tendency on the modified ERQ when adjusting for MCSD scores and age, *r*_*s*_(396) = .006, 95% CI [−.097, .11], *p* = .91. We also aimed to replicate findings from Study 1 indicating a relationship between COVID-related worry and suppression tendency. COVID-related worry was associated with greater use of suppression, as reported on the modified ERQ, when adjusting for MCSD and age, *r*_s_(396)= .10, 95% CI [.005, .21], *p* = .037.

#### Mediation Analysis

We attempted to replicate findings from Study 1 indicating a mediating role of COVID-related worry on the relationship between IUS and suppression tendency as measured by the modified ERQ. However, this preregistered analysis was not significant (*ab* = .040, 95% CI [−.030, .11], *p* = .27; proportion mediated = .16, *p* = .27).

## Discussion

Despite the impact of uncertainty on mental health, little is understood about how intolerance of uncertainty is associated with cognitive emotion regulation. Our replicated findings indicated that IUS scores were associated with greater worry about the pandemic and decreased capacity to use reappraisal. IUS was not significantly associated with emotional reactivity when adjusting for reappraisal capacity and was associated with greater tendency to use suppression.

We also hypothesized that COVID-related worry would mediate the relationships between IUS and emotion regulation. However, we observed inconsistent results as the mediating effect of COVID-related worry on the relationship between IUS and suppression tendency, observed in Study 1, was not replicated in Study 2. This may have been due to insufficient power, or perhaps differences across contexts in which a more protracted experience with the pandemic may have led to different mediating pathways in Study 2. For example, ongoing stressors in Study 2 may have led to increased social tension. A relevant factor may thus be rejection sensitivity, a construct that partly reflects intolerance for potential rejection in interpersonal situations (Downey & Feldman, 1996). However, we note these mediation analyses cannot assess causal relationships due to their correlational nature. In addition, the temporal proximity in which study measures were assessed could have led to biases. Although it is not possible to manipulate trait intolerance of uncertainty, future research could manipulate state intolerance of uncertainty, as well as worry, to assess their impact on emotion regulation. Manipulating intolerance of uncertainty as a state (Ladouceur et al., 2000) would also clarify how externally induced changes to intolerance of certainty — as opposed to the IUS, which measures subjective beliefs — may be associated with emotion regulation.

Additional caveats should be considered. First, our findings have limited generalizability as we only recruited participants from the United States, and we attempted to minimize the effects of age as a confound — as age was broadly associated with IUS, worry, and emotion regulation in Study 1. We adjusted for age in Study 1 and restricted recruitment for Study 2 to a young adult sample. Although we believe our findings are nevertheless of general interest — as young adults have been reported to be particularly vulnerable to experiencing emotional difficulties during the pandemic (O’Connor et al., 2021) — it would be informative to assess relationships between IUS and emotion regulation in developmental and aging populations. Second, although our research demonstrates that a commonly used reappraisal task can be adapted for online use — specifically, participants reported expected decreases in negative affect when using reappraisal as compared to responding naturally to negative stimuli — it is worth noting that the size of this effect was smaller than that typically observed in lab-based studies using variations of this task (e.g. Denny & Ochsner, 2014; Silvers et al., 2016). Further research will be needed to determine whether effect sizes consistently differ between online and lab-based populations in the context of studying emotion regulation. Finally, the reappraisal task we used in our studies assessed the ability to use reinterpretation to change the meaning of a situation. This, however, is only one kind of reappraisal tactic. Distancing, which involves taking a physically or temporally distanced perspective, is another reappraisal tactic demonstrated to have longer-lasting down-regulatory effects than reinterpretation (Denny & Ochsner, 2014). Thus, it may be that IUS exhibits different relationships with different kinds of reappraisal tactics. Speculatively, as the ERQ does not specifically assess reinterpretation, such differences may have contributed to null findings between COVID-related worry and reappraisal tendency assessed by the ERQ. It would be informative to investigate how IUS may be differentially associated with other kinds of reappraisal and emotion regulation strategies, as well as the regulation of autobiographical memories.

In sum, prior literature has documented that intolerance of uncertainty plays an important role in anxiety and affective disorders. Our studies indicate that trait intolerance of uncertainty is broadly associated with reappraisal and suppression, and specifically with impairments to top-down regulatory control of affect. As emotion dysregulation is associated with affective disorders (Campbell-Sills & Barlow, 2007; Cisler et al., 2010), assessing how intolerance of uncertainty is associated with emotion regulation is critical for understanding the mechanisms by which intolerance of uncertainty may impact mental health.

## Supplementary Information

ESM 1 (DOCX 36 kb)

## Additional Information

### Data Availability

Data and analysis scripts can be accessed at https://osf.io/kpf3d/. Preregistration for Study 2 can be viewed at https://aspredicted.org/zy7it.pdf.
