# Supplementary material for: Trait Intolerance of Uncertainty Is Associated with Decreased Reappraisal Capacity and Increased Suppression Tendency
Source: Affect Sci. 2022 Jun 3;3(3):528–38. doi: 10.1007/s42761-022-00115-8 (PMC9162878; doi:10.1007/s42761-022-00115-8)
Supplement: Supplementary file 1 — (DOCX 36 kb) [file 42761_2022_115_MOESM1_ESM.docx]

Trait Intolerance of Uncertainty is Associated with Decreased Reappraisal Capacity and Increased Suppression Tendency

Jocelyn Shu^1^, Kevin N. Ochsner^2^, & Elizabeth A. Phelps^1^

^1^Department of Psychology, Harvard University,

^2^Department of Psychology, Columbia University

Supplementary Online Materials (SOM)

This file contains exploratory analyses and Tables S1-S3.

**Exploratory Analyses for Study 1**

**Relationships between negative affect and reappraisal capacity.** Exploratory analyses were conducted to assess whether the PANAS, PSS, and DASS-21 were associated with reappraisal capacity on the task when adjusting for emotional reactivity (mean negative affect in Look Negative Condition), MCSD scores, and age. Partial correlation analyses indicated that PSS (*M* = 1.82, *SD* = .78; *r_s_*(158) = .11, 95% CI [-.041, .26], *p* = .18) and DASS-21 (*M* = .74, *SD* = .61, *r_s_*(158) = .057, 95% CI [-.096, .21], *p* = .47) were not significantly associated with reappraisal capacity when controlling for emotional reactivity**,** MCSD, and age. The negative scale of the PANAS (PANAS-Neg) was significantly associated with reappraisal capacity (*M* = 1.62, *SD* = .76, $\alpha$ = .93; *r_s_*(158) = .18, 95% CI [.030, .34], *p* = .020).

Although PANAS-Neg scores may reflect patterns of affective responding that overlap with the construct of intolerance of uncertainty, we used multiple linear regression to then assess the relationship between IUS and reappraisal capacity when adjusting for PANAS-Neg, along with emotional reactivity, MCSD, and age. Neither IUS (*b* = .075, 95% CI [-.037, .19], *SE* = .057, *t* = 1.33, *p* = .19, *f*^2^ = .011) nor PANAS-Neg (*b* = .088, 95% CI [-.021, .20], *SE* = .055, *t* = 1.60, *p* = .11, *f*^2^ = .016) was a significant predictor of reappraisal capacity.

**Mediation analysis.** Although we tested the mediating effect of COVID-related worry on the relationship between IUS and suppression tendency in the manuscript, it is also possible that suppression tendency mediates the relationship between IUS and COVID-related worry. An exploratory mediation analysis with bootstrapping using 10,000 samples, adjusting for MCSD scores and age, indicated the presence of a mediating effect for suppression tendency on the relationship between IUS and COVID-related worry (*ab* = .063, 95% CI [.005, .13], *p* = .031, proportion mediated = .14, *p* = .031).

**Exploratory Analyses for Study 2**

**Relationships between negative affect and reappraisal capacity.** Partial correlation analyses indicated that reappraisal capacity on the task was not significantly associated with the Affective Lability Scale (ALS; *M* = 2.10, *SD* = .68, $\alpha$ = .93; *r_s_*(395)= .084, 95% CI [-.020, .19], *p* = .095) or Penn State Worry Questionnaire (PSWQ; *M* = 3.22, *SD* = .97, $\alpha$ = .95; *r_s_*(395)= .092, 95% CI [-.005, .19], *p* = .068) when adjusting for emotional reactivity, MCSD scores, and age. However, as in Study 1, PANAS-Neg was significantly associated with reappraisal capacity when controlling for emotional reactivity, MCSD, and age (*M* = 1.56, *SD* = .72, $\alpha$ = .92; *r_s_*(395)= .13, 95% CI [.037, .23], *p* = .010). As in Study 1, we performed a multiple linear regression to assess the relationship of IUS with reappraisal capacity when adjusting for PANAS-Neg. Replicating findings from Study 1, neither IUS (*b* = .056, 95% CI [-.014, .12], *SE* = .035, *t* = 1.57, *p* = .12, *f*^2^ = .006) nor PANAS-Neg (*b* = .064, 95% CI [-.016, .14], *SE* = .041, *t* = 1.57, *p* = .12, *f*^2^ = .006) significantly predicted reappraisal capacity.

**Mediation analysis.** We attempted to replicate the result from the exploratory mediation analysis in Study 1 to assess whether suppression tendency mediates the relationship between IUS and COVID-related worry. Using the same analysis, the result in this study was not significant (*ab* = .009, 95% CI [-.008, .03], *p* = .27, proportion mediated = .026, *p* = .27).

Table S1

| *Items Administered to Assess COVID-Related Worry* | |
| --- | --- |
| 1. | How worried are you about the possibility that **you**might become infected with COVID-19? |
| 2. | How worried are you about the possibility that any of **your**close friends or family members might become infected with COVID-19? |
| How worried are you that in the future you will experience… | |
| 3. | ...decreased in-person visits to close friends or family? |
| 4. | ...decreased virtual contact with close friends or family (e.g., phone calls, texts, video chats, etc.)? |
| 5. | ...increased tension with people in your household? |
| 6. | ...increased tension with others outside of your household? |
| 7. | ...a loss of employment? |
| 8. | ...problems obtaining grocery items? |
| 9. | ...problems paying for bills (e.g., housing, food, medical bills)? |
| 10. | ...problems accessing healthcare? |
| 11. | ...problems receiving your usual paycheck? |
| 12. | ...difficulties combining childcare with work? |
| 13. | ...obstacles that make work more difficult? |
| 14. | ...an increased workload? |
| 15. | ...problems working from home? |
| 16. | ...decreased physical exercise? |
| 17. | ...decreased participation in your usual leisure activities? |
| 18. | ...a decrease in new fun activities? |
| 19. | ...boredom? |
| How worried are you that in the future... | |
| 20. | ...the government will not take appropriate action to combat issues surrounding COVID-19? |
| 21. | ...criminality rates will increase? |
| 22. | ...there will be an economic crisis? |
| 23. | ...other people will not follow public health guidelines to prevent the spread of COVID-19 (e.g., washing hands, social distancing, etc.)? |
| 24. | ...life as we knew it will never be the same? |

Notes. Items rated on a 5-point Likert scale (1 = Not at all worried, 3 = Somewhat worried, 5 = Extremely worried). In Study 1, items 3-24 included the option to select N/A. In Study 2, all items included the option to select N/A.

Table S2

| *Zero-Order Correlations Between Measures in Study 1* | | | | | | | | | | | |
| --- | --- | --- | --- | --- | --- | --- | --- | --- | --- | --- | --- |
| Measure | Reappraise (Task) | Look Neg (Task) | ERQ (Reapp) | ERQ (Supp) | IUS | COVID Worry | MCSD | Age | PANAS (neg) | PANAS (pos) | PSS |
| Reappraise (Task) |  |  |  |  |  |  |  |  |  |  |  |
| Look Neg (Task) | .69*** |  |  |  |  |  |  |  |  |  |  |
| ERQ (Reapp) | -.05 | .11 |  |  |  |  |  |  |  |  |  |
| ERQ (Supp) | .00 | -.01 | -.12 |  |  |  |  |  |  |  |  |
| IUS | .33*** | .25** | -.18* | .32*** |  |  |  |  |  |  |  |
| COVID Worry | .26*** | .22** | -.02 | .26*** | .41*** |  |  |  |  |  |  |
| MCSD | -.13 | -.07 | .16* | -.01 | -.37*** | -.23** |  |  |  |  |  |
| Age | -.27*** | -.16* | .19* | -.15 | -.20* | -.18* | .21** |  |  |  |  |
| PANAS (Neg) | .39*** | .34*** | -.22** | .27*** | .49*** | .53*** | -.27*** | -.24** |  |  |  |
| PANAS (Pos) | .02 | .17* | .40*** | -.24** | -.15 | -.12 | .28*** | .32*** | -.19* |  |  |
| PSS | .16* | .05 | -.31*** | .30*** | .64*** | .51*** | -.34*** | -.29*** | .57*** | -.45*** |  |
| DASS-21 | .20** | .16* | -.28*** | .38*** | .60*** | .55*** | -.37*** | -.27*** | .67*** | -.36*** | .81*** |

Notes. Spearman’s rho correlation coefficients are displayed. Reappraise (Task) = mean negative affect in Reappraisal Condition of reappraisal task, Look Neg (Task) = mean negative affect in Look Negative Condition of reappraisal task, ERQ (Reapp) = reappraisal subscale of the Emotion Regulation Questionnaire, ERQ (Supp) = suppression subscale of the Emotion Regulation Questionnaire, IUS = Intolerance of Uncertainty Scale, COVID Worry = 24-item measure of COVID-related worry, MCSD = Marlowe Crowne Social Desirability Scale short form, PANAS (Neg) = negative subscale of the Positive and Negative Affective Schedule, PANAS (Pos) = positive subscale of the Positive and Negative Affective Schedule, PSS = Perceived Stress Scale, DASS-21 = Depression, Anxiety, and Stress Scale. **p* < .05, ***p* < .01, ****p* < .001

Table S3

| *Zero-Order Correlations Between Measures in Study 2* | | | | | | | | | | | | |
| --- | --- | --- | --- | --- | --- | --- | --- | --- | --- | --- | --- | --- |
| Measure | Reappraise (Task) | Look Neg (Task) | ERQ (Reapp) | ERQ (Supp) | IUS | COVID Worry | MCSD | Age | PANAS (neg) | PANAS (pos) | PSS | PSWQ |
| Reappraise (Task) |  |  |  |  |  |  |  |  |  |  |  |  |
| Look Neg (Task) | .73*** |  |  |  |  |  |  |  |  |  |  |  |
| ERQ (Reapp) | .05 | .06 |  |  |  |  |  |  |  |  |  |  |
| ERQ (Supp) | -.06 | -.04 | .02 |  |  |  |  |  |  |  |  |  |
| IUS | .16** | .10* | -.20*** | .13** |  |  |  |  |  |  |  |  |
| COVID Worry | .24*** | .22*** | -.01 | .10* | .37*** |  |  |  |  |  |  |  |
| MCSD | -.01 | .02 | .14** | .02 | -.31*** | -.09 |  |  |  |  |  |  |
| Age | -.03 | .00 | .03 | -.03 | -.12* | .03 | .07 |  |  |  |  |  |
| PANAS (Neg) | .19*** | .13* | -.23*** | .15** | .46*** | .30*** | -.22*** | -.15** |  |  |  |  |
| PANAS (Pos) | .10* | .22*** | .37*** | -.05 | -.16** | .02 | .22*** | .16** | -.16** |  |  |  |
| PSS | .14** | .09 | -.29*** | .11* | .57*** | .39*** | -.33*** | -.18*** | .55*** | -.36*** |  |  |
| PSWQ | .13** | .08 | -.25*** | .03 | .70*** | .36*** | -.30*** | -.14** | .46*** | -.33*** | .65*** |  |
| ALS | .12* | .08 | -.17*** | -.01 | .57*** | .33*** | -.35*** | -.15** | .49*** | -.17*** | .63*** | .55*** |

Notes. Spearman’s rho correlation coefficients are displayed. Reappraise (Task) = mean negative affect in Reappraisal Condition of reappraisal task, Look Neg (Task) = mean negative affect in Look Negative Condition of reappraisal task, ERQ (Reapp) = reappraisal subscale of the Emotion Regulation Questionnaire, ERQ (Supp) = suppression subscale of the Emotion Regulation Questionnaire, IUS = Intolerance of Uncertainty Scale, COVID Worry = 24-item measure of COVID-related worry, MCSD = Marlowe Crowne Social Desirability Scale short form, PANAS (Neg) = negative subscale of the Positive and Negative Affective Schedule, PANAS (Pos) = positive subscale of the Positive and Negative Affective Schedule, PSS = Perceived Stress Scale, PSWQ = Penn State Worry Questionnaire, ALS = Affective Lability Scale. **p* < .05, ***p* < .01, ****p* < .001
